# JAK inhibitors: a potential treatment for JDM in the context of the role of interferon-driven pathology

**DOI:** 10.1186/s12969-021-00637-8

**Published:** 2021-09-25

**Authors:** Meredyth G. Ll Wilkinson, Claire T. Deakin, Charalampia Papadopoulou, Despina Eleftheriou, Lucy R. Wedderburn

**Affiliations:** 1 Infection, Immunity and Inflammation Programme Research and Teaching Department, UCL Great Ormond Street Institute of Child Health, University College London, 30 Guilford Street, London, WC1N 1EH UK; 2 Centre for Adolescent Rheumatology Versus Arthritis at UCL UCLH and GOSH, University College London, London, UK; 3 NIHR Biomedical Research Centre at GOSH, London, UK; 4 Rheumatology, Great Ormond Street Hospital, Great Ormond Street, London, UK

**Keywords:** Juvenile dermatomyositis, JDM, IIM, JAK inhibitors, IFN, JAK/STAT pathway, Treatment

## Abstract

Juvenile Idiopathic Inflammatory Myopathies (IIM) are a group of rare diseases that are heterogeneous in terms of pathology that can include proximal muscle weakness, associated skin changes and systemic involvement. Despite options for treatment, many patients continue to suffer resistant disease and lasting side-effects. Advances in the understanding of the immunopathology and genetics underlying IIM may specify new therapeutic targets, particularly where conventional treatment has not achieved a clinical response. An upregulated type I interferon signature is strongly associated with disease and could be a prime target for developing more specific therapeutics. There are multiple components of the IFN pathway that could be targeted for blockade therapy.

Downstream of the cytokine receptor complexes are the Janus kinase-signal transducers and activators of transcription (JAK-STAT) pathway, which consists of JAK1–3, TYK2, and STAT1–6. Therapeutic inhibitors have been developed to target components of this pathway. Promising results have been observed in case studies reporting the use of the JAK inhibitors, Baricitinib, Tofacitinib and Ruxolitinib in the treatment of refractory Juvenile Dermatomyositis (JDM). There is still the question of safety and efficacy for the use of JAK inhibitors in JDM that need to be addressed by clinical trials. Here we review the future for the use of JAK inhibitors as a treatment for JDM.

**Supplementary Information:**

The online version contains supplementary material available at 10.1186/s12969-021-00637-8.

## Introduction

Idiopathic Inflammatory Myopathies (IIM) are a group of rare immune-mediated diseases that are heterogeneous in terms of pathology, clinical phenotypes and age of onset (Table 1). JDM is very rare with an annual incidence of three cases per million children [2, 23, 24] and median age of onset 6.3 years old (IQR; 3.8–9.6) [1]. Children typically present with symmetrical proximal and axial muscle weakness and characteristic skin changes including Gottron’s papules and heliotrope rash. Long-term complications include lung fibrosis, lipodystrophy and calcinosis [25–29] . In most JDM cohorts, 60–70% of children with JDM are positive for an autoantibody [30–33]. A number of myositis specific antibodies (MSA) have been described associated with a variety of phenotypes in JDM [10].

**Table 1:** JDM disease features

**Epidemiology**	Median age of onset (IQR):	6.3 (3.8–9.6) years [1]
	Incidence:	7.98 cases/million/year [2]
	Prevalence:	14/100,000 [2]
	Sex distribution (F:M):	2.1:1 [3]
**Clinical features**	Muscle weakness	Most patients
	Cutaneous manifestations	30–70% [3]
	Calcinosis	12–47% [4, 5]
	Lipodystrophy	8–14% [6]
	Interstitial lung disease	8–19% [7]
	Myocardial involvement	Common, non-specific [8]
	Vasculopathy	Most patients, central to pathogensis [9]
**Autoantibodies**	MSA 49% + ve for MSA	- Transcriptional intermediary factor 1 (TIF-1γ) 22–29%
		- Nuclear matrix protein 2 (NXP2) 23–25%
		-Aminoacyl tRNA synthetase (ASA) 2–4%
		-Signal recognition particle (SRP) < 2%
		−3-hydroxy-3-methylglutaryl-coenzyme A reductase (HMGCR) < 1%
		-Nucleosome-remodelling deacetylase complex (Mi-2) 4–10%
		-Small ubiquitin-like modifier activating enzyme (SAE) < 1%
		-Melanoma differentiation associated gene 5 (MDA5) 7–38% [10]
**Pathogenesis**	Type I IFN signature	Muscle, blood [11, 12]
	Mononuclear cells	Muscle [15]
	FOXP3+ regulatory T cells	Increased in muscle [16]
	pDCs	Increased in muscle/skin [17]
	Myogenic pre-cursor cells	Increased source of IFN in muscle [18, 19]
	Mast cells	Increased in skin [20]
	Natural killer cells	Decreased in blood [21]
	Cytokines	
	Blood:	Increased IRF-4, IL-6, IL-17F, Il-23A, IL-21, GATA3, IL-1β
	Muscle:	Increased GATA3, IL-13, STAT5B [22]

## The need for new treatments

The mainstay treatments for IIM are prednisolone and methotrexate, and even those patients who respond well to these drugs can have prolonged disease [34, 35]. Other immunotherapy treatments used include mycophenolate mofetil, cyclophosphamide, intravenous immunoglobulin (IvIG), azathioprine, cyclosporine and tacrolimus [36–38]. Biological targets include blockade of tumour necrosis factor alpha (TNFα) and B cells (anti-CD20). As potential treatments for JDM, efficacy was reported in a case series of the use of adalimumab and infliximab (TNFα blockades), and also in an International study of B cell depletion by rituximab (anti-CD20) [39, 40]. However, there is a need for more targeted treatments and methods to identify patients who will require these.

Several more recent emerging biologic therapies for the treatment of IIM have been reported including; belimumab, abatacept, bimagrumab, spiponimod, apremilast, gevokizumab, eculizumab and basiliximab (Table 2) [41–48]. Sifalimumab, is a fully human immunoglobulin G_1_ κ anti-IFNα monoclonal antibody that binds to and neutralizes the majority of IFN-α subtypes, is an important candidate therapeutic due to the wealth of evidence of the strong IFN signature identified in myositis [11, 12, 50–55]. A phase 1b clinical trial of sifalimumab in adult patients with dermatomyositis (DM) and polymyositis (PM), used outcome measures of IFN gene signature suppression against disease improvement. Initial results suggested that targeting the IFN pathway with sifalimumab showed more neutralisation of IFN gene expression in patients that had greater improvement of disease, but blockade of the type I IFN receptor (IFNAR) may offer superior clinical benefit [49]. Beyond the therapeutics highlighted in Table 2 there are potential new therapies for the treatment of IIM including JAK inhibitors to target the IFN pathway.

**Table 2:** Emerging biologic therapies for the treatment of adult and juvenile IIM

Biologic	Mechanism	Clinical trial type	Clinical trial number	Patient group	Outcome
rituximab [39]	Monoclonal anti-CD20 antibody that depletes B cells	Randomized, double-blind, placebo-phase trial	NCT00106184	JDM and DM	Higher proportion of JDM (87%) patients treated with rituximab met the definition of improvement more quickly compared to adult DM (78%)
belimumab	Anti-B cell activating factor (BAFF) monoclonal antibody	Multicentre double-blind, placebo-controlled trial	NCT02347891	Refractory IIM	Evaluating the efficacy and safety
abatacept	Modified fully human soluble recombinant protein that consists of cytotoxic T cell lymphocyte antigen-4 (CTLA4) fused with Fc region of human IgG1	Interventional clinical trial	NCT02594735 NCT03215927 NCT02971683	Refractory JDM Myositis-associated ILD IIM	Clinical improvement Evaluate efficacy and safety
bimagrumab [41, 42]	Human recombinant monoclonal anti-ACVR2B activin type 2 receptor antibody	Phase IIb/III double-blind, placebo-controlled multicentre study Phase IIb/III Study	NCT01925209 CBYM338B2203	IBM/IIM	Improvement in muscle volume and strength
spiponimod	Oral selective sphingosine-1-phosphate receptor modulator, acts by preventing the migration of lymphocytes to inflammatory sites and therefore reducing inflammation	Multicentre, phase 2, double-blind, randomized, controlled trial	NCT02029274NCT01148810	IIM	International Myositis Assessment Study (IMACS) definition of improvement
apremilast [44]	Phosphodiesterase-4(PDE-4) inhibitor, reduces the expression of pro-inflammatory cytokines by increasing cyclic adenosine monophosphate	Open-label, single-centre study Phase two, open-label, single group assignment, interventional study	NCT01140503, NCT03529955	DM	30% reduction in the cutaneous disease activity and severity index (CDASI) Safety, efficacy and clinical response
gevokizumab	Humanised IgG2 monoclonal antibody against human IL-1β	Proof-of-concept, randomized, double-blind, placebo-controlled trial	EudraCT number: 2012–005772-34	IIM	Prematurely terminated therefore limited results
eculizumab [46, 47]	Monoclonal humanised antibody against terminal complement components	Randomized, double-blind, placebo-controlled pilot study Phase two, randomized, placebo-controlled, third-party-blind study	NCT00005571	IIM DM	Improvement of global physician score for cutaneous disease Evaluation of safety and efficacy, results pending.
basiliximab [48]	IL-2R chimeric monoclonal antibody; blocks Il-2 receptor on the surface of activated T-cells	Open-label, randomized, parallel assignment without masking, phase-2, single center study	NCT03192657	Amyopathic dermatomyositis (CADM) patients with interstitial pneumonia	Primary outcome measure is survival at 52 weeks
sifalimumab [49]	anti-IFNα monoclonal antibody	Double-blind, phase 1b multicentre randomized control trial	NCT00533091	DM and PM	Neutralisation of IFN gene signature suppression against disease improvement

## Interferon: mechanisms in autoimmune disease

While the interferon family are a group of molecules central to the anti-viral responses, many autoimmune diseases also have an aberrant interferon response. Gene activation is the main mechanism for the interferon anti-viral response, but interferons are also integral to intra-cellular signalling in the immune system (Additional file 1: Supplementary Fig. 1 [56]). Many autoimmune diseases have been found to have an up-regulated IFN type I signature, including systemic lupus erythematous (SLE), rheumatoid arthritis (RA) and myositis [11, 50, 51, 57–59]. The IFN type I comprise of thirteen types including IFN-α, IFN-β, IFN-κ, IFN-ω and IFN-ν; these bind to a common receptor, IFN-α receptor (IFNAR), but the differences in induction of cellular responses is poorly defined [60]. There are three proposed mechanisms. The first is that plasmacytoid dendritic cells (pDCs) are activated by endogenous IFN inducers to produce IFN-α [61]. The second is that genes associated with autoimmune disease risk, lie within the IFN type I signalling pathway that in turn effect the production and response of IFN-α. IFN-regulatory factor (IRF) 5 was identified as a SLE risk gene as it has increased expression and is activated in SLE patients [62–64]. Other autoimmune diseases have specific risk genes that associate with the IFN signature [65]. The third mechanism proposes that regulation and control of plasmacytoid dendritic cells (pDC) and the expression of interferon regulatory genes (IRG) is not functioning correctly [61]. A decrease in reactive oxygen species (ROS) production from monocytes can lead to enhanced autoimmunity. In addition there is a predominant STAT1 signature in ROS deficient disease [66]. The relative contribution of these three mechanisms may differ between autoimmune disease, severity and patient.

## Role of interferons in myositis

The most abundant IFN type I are IFN-α and IFN-β. The IFNs bind to the IFN-α receptor (IFNAR) and activate the Janus kinase (JAK)-signal transducer and transcription (STAT) pathway that in turn lead to the transcription of IFN-stimulated genes (ISGs) [67, 68]. The over production of IFN in the blood and muscle is an abnormality in the pathogenesis of dermatomyositis [13, 14, 69]. The release of IFN type I leads to immune cell activation and vasculopathy. A major source of IFN type I is from plasmacytoid dendritic cells (pDC) after activation by either self-DNA or viral nucleic acid [70, 71]. Plasmacytoid dendritic cells (pDC) have been identified in JDM muscle, but IFN type I is difficult to detect in serum due to limits of sensitivity of existing assays until recently [11, 72]. The Simoa assay developed by Rodero et al. can detect IFN-α at differential levels and determine cellular sources measured from lysed cell-subsets [57]. Using this assay IFN-α levels were significantly increased in sera from a JDM cohort compared to a healthy cohort [57, 73].

Due to the difficulties in measuring IFN directly, gene expression is often used as a marker of the activation of the IFN type I pathway. An IFN score was developed to encompass a selection of the IFN response genes, *IFI27*, *IFI44L*, *IFIT1*, *ISG15*, *RSAD2* and *SIGLEC1*, these are measured by quantitative reverse transcription polymerase chain reaction (qPCR) [74]. Other studies have also measured expression of additional genes including ISG15 ubiquitin-like modifier (*G1P2*), and interferon regulatory factor 7 (*IRF7*) [51, 71]. Variations of this score have been used to correlate with disease in multiple studies [53, 75, 76]. A signature of 43 genes was elevated in myositis compared to controls [14]. A positive correlation has been shown between an IFN score (6 genes) compared to serum IFN-α levels (*n* = 24, R_s_ = 0.620, *p* = 0.0004) taken from JDM patients [57]. The type II IFN signature also correlates to disease activity in JDM and other chemokines [77]. This suggests that as a whole the IFN family are upregulated in the context of JDM and adult DM. The clinical trial of sifalimumab in DM/PM showed suppression of the IFN gene signature in blood and muscle tissue of the IIM patient cohort. Patients with 15% or greater improvement from baseline manual muscle testing scores (MMT8) showed greater neutralisation of the interferon gene signature than patients with less than 15% improvement [49]. This trial highlights the potential for the therapeutic targeting of interferon in DM and JDM.

Another indirect measure is the IFN-driven protein signature which may include measurement of levels of monocyte chemoattractant protein 1 (MCP-1), monocyte chemoattractant protein 2 (MCP-2), interferon gamma-induced protein 10 (IP-10), tumour necrosis factor receptor II (TNFRII), galectin 9 and chemokine (C-XC motif) ligand 9 (CXCL9). These proteins, measured in serum, significantly correlated with disease activity in JDM [22, 78–80]. Chemokines and cytokines have shown to correlate with the IFN signature in peripheral blood mononuclear cells. A study in JDM showed an expansion of peripheral blood naïve immature B cells, skewed to an inflammatory profile, in early disease, that correlated with an IFN type I score taken from RNA-seq analysis of B cells and downstream IFN proteins [81].

Circulating endothelial cells (CEC) have been detected in peripheral blood and associated with vascular injury [82]. An in vitro study has shown that IFN type I treatment of HUVECs impaired endothelial cell function, with significant reduction of tubule formation when HUVECs were cultured with IFN type I + VEGF and anti-IP10 [83]. A recent study in JDM, identified higher CEC in both active and definite inactive disease (JDM *n* = 90; median 96(IQR 40–192) cell/ml compared to controls *n* = 79; median 12(IQR 8–24) cells/ml, *p* < 0.0001). They also showed a strong correlation with other markers of vascular injury including endothelial microparticles and galectin-9 [9]. Another study showed that CEC correlated with extra muscular disease activity but not muscular damage [84]. In JDM, CEC may prove to be a useful biomarker for underlying disease pathology.

Key sites of inflammation in JDM are the muscle and skin. Both muscle and skin tissue biopsy material can provide valuable insights to our understanding of an individual JDM patients disease [85–88]. These tissue samples are the key to understanding the pathophysiology of disease at the tissue site. IFN type I and other cytokines have been detected within the inflamed muscle [89]. The IFN proteins (IFN-α,-β,-γ) themselves have been detected in muscle, but also the IFN-stimulated proteins ISG15, MxA and class I MHC [90–92]. Higher levels of ISG15 were quantified in JDM muscle tissue compared to non-JDM [93]. Markers of disease activity and muscle damage have been shown to correlate with the expression of MxA in the muscle tissue [94, 95]. Research has been carried out to identify the direct effects that IFN type I has on muscle tissue types. Muscle atrophy and loss of myogenin has been detected on muscle myotubes, reduced junctions and capillary growth on endothelium [96]. A recent study has shown that these effects have been blocked in vitro by the JAK inhibitor Ruxolitinib [97]. These findings build a picture of the interferonopathy at both the tissue site and the peripheral blood.

## The JAK-STAT pathway – a therapeutic target

**Fig. 1:**
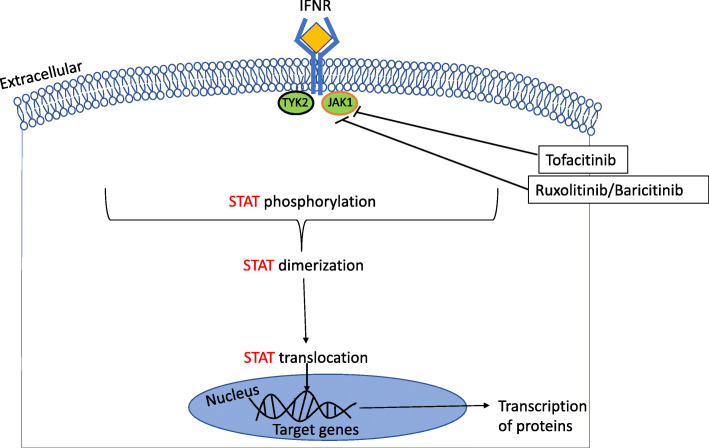
JAK-STAT pathway with JAK inhibitor targets. The activation of the JAK-STAT pathway after IFN type 1 has engaged with the associated receptor, IFNR. This induces the transcription of proteins. Tofacitinib inhibits JAK1/2/3. Ruxolitinib and Baricitinib inhibit JAK 1/2, inhibtion prevents STAT phosphorylation, dimeraziation and transolcation into the nucleuse. This in turn stops the transcription of pro-inflammatory proteins

When IFN binds to its respective receptor, IFN-R, on the cell surface membrane, this in turn activates the signalling cascade inducing the Janus kinase-signal transducers and activators of transcription (JAK-STAT) pathway [98, 99] (Fig. 1). The JAK-STAT pathway consists of JAK1–3, TYK2, and STAT1–6, of these JAK1 and TYK2 are directly activated by IFN type 1 proteins. This signalling cascade triggers the receptor-associated JAK to phosphorylate the receptor and other JAKs [100]. If specific tyrosine motifs are phosphorylated in the cytokine receptor, then a docking-site for STATs is opened enabling further phosphorylation of STATs. When STATs are phosphorylated they dimerize through their Src homology domain-2 (SH2) domains, this allows them to translocate to the nucleus and activate specific genes [101]. An individual receptor is made up of several subunits, each is associated with a specific JAK. Therefore, each receptor chain can have more or less specificity to an individual JAK. The JAK-STAT pathway could offer a potential target for the blockade of the transcription of IFN genes [100].

## JAK inhibition

**Fig. 2:**
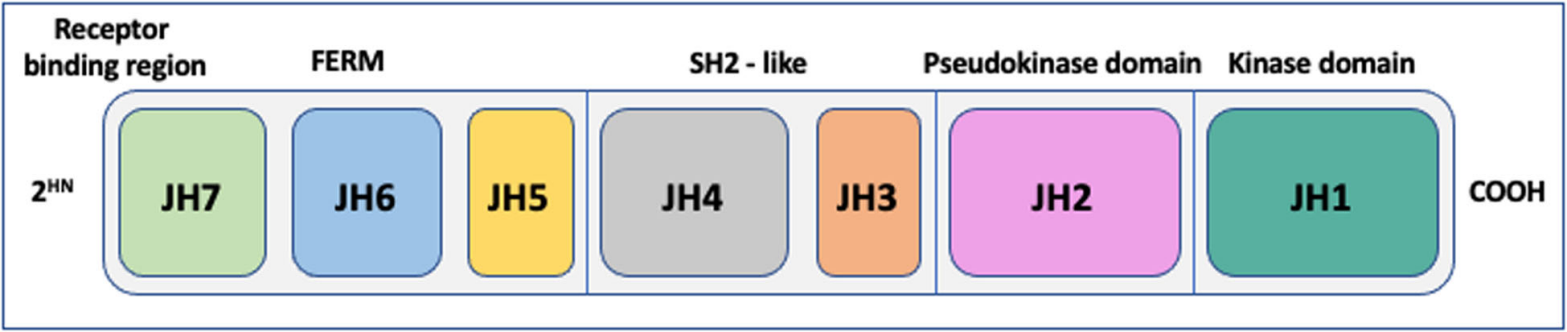
JAK domains and homologous regions. JAKs are constructed from four domains made of seven homologous regions (JH1–7)

JAKs are constructed from four domains made of seven homologous regions (JH1–7) (Fig. 2). To date JAK inhibitors (JAK-inhibitors) have generally targeted the JH1 domain. JH1 is the active catalytic phosphotransferase domain and competes with adenosine triphosphate at the catalytic site [102]. JH2 is a pseudokinase domain that supresses ligand-independent kinase activity, the mode of action is direct interaction with JH1 and activation of ligand-induced JAK [103]. Deucravacitinib is an example of a JAK inhibitor that targets the JH2 psuedokinase domain [104, 105]. JH3/4 have a primary role in stabilising the structure of the enzyme. JH5–7 associate JAKs with their cognate receptors [106]. There have been multiple JAK-inhibitors that have been or are in development. These can be defined in two categories; first-generation or next-generation JAK-inhibitors [100]. The first-generation exert pan-inhibition on all four of the JAKs, these include; tofacitinib, ruxolitinib, baricitinib, and oclacitinib [107]. The next-generation of JAK-inhibitors are more specific in their target blockade, these include; fedratinib, momelotinib, and pacritinib [108]. This specificity should help with disease targeted treatment and reduce associated side-effects.

Inhibition of TYK2 is an example of a more specific next-generation Jakinib. TYK2 has been associated with several autoimmune conditions including; RA, JIA, SLE, type 1 diabetes and MS [109–114]. A GWAS analysis of IIM in Caucasian individuals identified that a non-synonymous SNP rs2304256 in TYK2 was associated with DM, IIM but not PM (Bonferroni correction *p* = 0.17, [115]. In a study of a Chinese Han population, analysis of TYK2 SNPS associated with DM and PM excluded TYK2 rs2304256 as it deviated from the Hardy-Weinberg equilibrium (HWE) in healthy controls [116]. This SNP is in the protein FERM (4.1 protein, erzin, radixin, moesin) domain, mediating interaction with JAK and microtubule interacting proetin1, this is thought to be increased in DM. Examples of TYK2 inhibitors trialled in psoriasis include; brepocitinib, BMS-986165 and PF-06826647 [117]. TYK2 is just one proposed target for inhibition in IIM.

## JAK inhibitor use in IIM – clinical trials

For the potential treatment of autoimmune conditions, multiple JAK inhibitors have been developed, trialled or approved [104, 107]. In adults the metabolism, pharmacokinetics and efficacy of JAK-inhibitors are highlighted in Additional file 1: Supplementary Table 1. Clinical trials are ongoing to determine the safety and efficacy of the use of multiple JAK-inhibitors as a therapy for treatment-resistant adult IIM. In addition to their small open-label, proof-of concept study of tofacitinib in 10 treatment-resistant DM patients (6 were anit-TIF1-γ positive), Paik et al. are carrying out a larger randomised controlled trial, with results pending (NCT03002649). Initial results from 10 participants showed they all met the primary outcome DOI at 12 weeks, 5 of 10 (50%) had moderate improvement and 5 of 10 (50%) had minimal improvement according to the 2016 ACR/EULAR myositis response criteria. The secondary outcome showed a significant change in CDASI disease activity score (mean average 28 ± 15.4 (baseline) vs. 9.5 ± 8.5 (12 weeks), *p* = 0.0005). There was also a trend towards a reduction of CXCL9/10 in serum and STAT1 signalling in 3 of 9 skin biopsies [118]. Another case report of 3 patients with refractory DM and calcinosis treated with tofacitinib showed an improvement of their calcinosis after 12 weeks (3 months) on treatment [119]. There is little know about the pathology of calcinosis but if JAK-inhibitors are effective then the JAK-STAT pathway may play a role in the underlying mechanism. Chen et al. are conducting a single centre, open-label clinical study of the use of tofacitinib in amyopathic dermatomyositis-associated ILD (Chinese Clinical Trial Registry number, ChiCTR-1,800,016,629). Initial results showed that 26 week (6-month) survival after onset of ILD was significantly higher in the prospective group (18 of 18, 100%) compared to the historical controls (25 of 32, 78%, *p* = 0.04), more conclusive results are pending [120]. Another ongoing study of the use of Baricitinib in adult IIM, is the MYOJAK study, a phase II, multicentre, randomised treatment delayed-start trial to receive active treatment (Baricitinib) or delayed-start after 13 weeks (NCT04208464). These trials are currently only including adult IIM patients of which have different clinical features to that of juvenile disease. Children have distinct developmental and physiological differences to adults, as such it is important to test the pharmokinectics and formulation of any given drug in the appropriate age populations.

## Evidence for the use of JAK inhibitors in JDM

There have been several reports and case series which support the need to pursue testing JAK-inhibitors for the future therapeutic use in juvenile DM (Table 3). The potential for Ruxolitinib was shown in a report of compassionate treatment for a case of severe vasculopathic refractory JDM. The thirteen year old patient presented with severe disease and was admitted to ICU after 3 weeks of diagnosis with multi-symptom, systemic disease. Over a period of 78 weeks (18 months) the patient was poorly controlled with combination therapy, and developed lower limb oedema and diffuse fascia calcinosis. The IFN type I signature was investigated, which showed IFN-α serum levels and IFN score were increased compared to controls, this was also the case with constitutive phosphorylation of STAT1/3 in T-cells and monocytes. From these results the patient was taken off MMF, rituximab infusions were stopped, and switched to Ruxolitinib (10 mg BD) with Prednisolone. After 2 table there was a noted improvement in disease activity scores and no reported adverse events. During the 52 weeks (12 months) of Ruxolitnib treatment the IFN measures did not normalise, but there was decreased STAT 1 phosphorylation in T cells [121].

**Table 3:** Case studies or case series of JAK-inhibitors in juvenile dermatomyositis

Case study	JAK-inhibitors	Patient	Disease course and prior treatment	Outcome
Aeschilimann et al. 2018 [121]	ruxolitinib	13 year old; JDM (anti-NXP2)	- Un-controlled disease with admission to ICU - Complexity of severe symptoms over 18 months -Prednisolone dependant, refractory to treatment including; methotrexate, IVIG, plasma exchange, MMF and rituximab -Increased IFN scores and STAT1 phosphorylation of T-cells and monocytes	After 52 weeks (12 months) of treatment: -Improvement of disease activity scores - decreased STAT1 phosphorylation in T-cells
Papadopoulou et al. 2019 [122]	baricitinib	11 year old; JDM (anti-TIF1-γ, anti-Ro52)	- 7 year history of JDM (with calcinosis) - steroid dependant; refractory to sequential treatment with azathioprine, mycophenolate mofetil, infliximab, adalimumab, rituximab, tacrolimus and cyclosporine, intravenous immunoglobulin (IVIG) - negative for class 4 and 5 variants of monogenic interferonopathies	After 26 weeks (6 months) of treatment: - clear improvement of disease - IFN biomarkers decreased - decreased level of CEC
Sabbagh et al. 2019 [123]	tofacitinib	2 anti-MDA5 JDM patients 12y/o male 15y/o female	Elevated 28-gene IFN score Un-controlled disease: Patient 1 – continuous flares after treatment with pulsed methylprednisolone, IVIg, methotrexate, MMF, rituximab Patient 2 – continuous flares after treatment with pulsed methylprednisolone, IVIg, MMF, abatacept, cyclophosphamide, rituximab and sildenafil	After 26 week (6 months) of treatment: - decrease in disease activity score - Decrease of IFN score and STAT1 phosphorylation of T-cells and monocytes
Yu et al. 2020 [124]	tofacitinib	n = 3 JDM 11y/o fem (ANA 1:320, anti-MDA5) 10y/o female (ANA 1:80, anti-Mi-2, anti-Ro-52 10y/o male (Negative)	Refractory JDM: patients failed ≥2 steroid sparing agents or high-dose steroids.	After 26 week (6 months): - Significant improvement of clinical scores; CMAS, MMT8, PGA, DAS and CHAQ
Le Voyer et al. 2021 [125]	baricitnib ruxolitinib	*n* = 3 JDM 2/3 female mean 8.7 years [25–30] NXP2 = 1 TIF1-y = 1 MDA5 = 1 No MSA = 0 *n* = 7 JDM 5/7 female mean 9.1 years [1, 2, 25–33] NXP2 = 3 TIF1-y = 2 MDA5 = 1 No MSA = 1	9 refractory disease and 1 new-onset Refractory muscle involvement (*n* = 8) Ulcerative skin disease (*n* = 2)	After 26 weeks (6 months): →Improvement in clinical scores →Clinically inactive disease →Decrease in seral IFN-α
Ding et al. 2021 [126]	tofacitinib 7/25 (28%) ruxolitinib 18/25 (72%)	*n* = 25 JDM 11/25 (44%) female Mean age of onset 4.6 ± 3.3 years Mean age to start JAK inhibitors 7.2 ± 4 years	All refractory 8/25 (32%) ineffective treatment 17/25 (68%) glucocorticoid dependant	25 patients followed up median of 34 weeks (7 months) (range – 3-21 months) →24/25 (96%) had rash improvement, 16/24 (66.7%) complete resolution →7/25 (28%) improved CMAS
Kim et al. 2021 [127]	baricitinib	4 JDM (5.8–20.7 years old)	→Chronically active disease →Failed 3–6 immunomodulatory medications	After 24 weeks of treatment: →Disease improvement assessed by clinical score →Down regulation of IRG →Decrease in serum IP-10

Positive results were seen in a compassionate case of the use of the Jakinib, Baricitinib for an eleven year old male with a seven year history of refractory JDM positive for anti-TIF1-γ and anti-Ro52 autoantibodies. When Baricitinib therapy was started clear improvement of disease was recorded. The IFN biomarkers, IFN type I signature and STAT1 phosphorylation in T cells and monocytes, decreased to comparative levels seen in controls. Also observed was a marked decrease of CEC. To note this was a singular, very severe case, however for the first time in seven years prednisolone could be tapered down, progression of calcinosis was halted and the disease improved as a whole [122]. Further prospective studies need to be carried out to investigate the safety and efficacy of Baricitnib for the use in the treatment of JDM.

A report of 2 patients with anti-MDA5 AB+ JDM with uncontrolled disease were treated with tofacitinib. Disease activity scores decreased within 26 weeks (6 months) following the start of tofacitinib therapy; IFN score, STAT1 phosphorylation of T-cells and monocytes decreased. This report shows evidence that tofacitinib improves JDM at an immunopathogenic level [123]. Another recent report of 3 cases of refractory JDM showed that 26 weeks (6 months) of treatment with tofacitinib was tolerated and the patients responded well to the treatment. Comparing 0–26 weeks (0–6 months) on treatment there were significant improvements in physician global VAS (*p* < 0.001), manual muscle testing-8 (MMT) (*p* = 0.002), child myositis assessment scale (CMAS) (*p =* 0.006), C-HAQ (p < 0.001) and DAS (*p =* 0.002). This set of case reports showed that tofacitinib treatment improved signs and symptoms of JDM and could be a promising treatment option [124].

A recent retrospective study included nine refractory and one new-onset JDM patients treated with ruxolitinib (*n* = 7) or baricitinib (*n* = 3). At 26 weeks (6 months) of follow up five of the ten patients (three Ruxolitnib and two Baricitinib) had reached clinically inactive disease (CID). In these patients the mean daily dose of steroids decreased from 1.1 mg/kg (range 0.35–2) to 0.1 (range, 0–0.3, *p* = 0.008). Serum IFN-α levels normalised 26 weeks (6 months) after the start of treatment in all patients [125].

A larger case series of refractory JDM patients, 8/25(35%) treatment was ineffective and 17/25 (68%) glucocorticoid dependant, were treated with tofacitinib 7/25(28%) or ruxolitinib 18/25 (72%). All 25 patients were followed up for a median of 30 weeks (7 months) (range = 3–21 months). 24/25 (96%) of patients had improvement of their rash of which 16/24 (66.7%) the rash completely resolved. The cutaneous assessment tool binary method score significantly decreased (7.0(3.0–10.0) to 0.0(0.0–1.0) *p* < 0.001). As a measure of muscle activity 7/25 (28%) of patients showed an improvement of CMAS score (from 18.6 ± 15.0 to 35.7 ± 6.3, *p* = 0.018). As of follow-up in August 2019 7/25 (28%) of patients had discontinued glucocorticoids. This case series has shown promise for the use of both drugs especially to improve skin disease [126].

Recently data has been published from a compassionate use study (NCT01724580) for the treatment of JDM with Baricitinib. Four JDM patients with chronically active disease were assessed at regular intervals over a 24 week period. There was significant improvement in clinical scores from 4 weeks (Physicians Global Assessment, Pt Global activity and CDASI activity score) and down-regulation of IRG score (28 genes) and serum IP-10. In CD4+ and CD8+ T Cells there were lower levels IFN-α stimulated pSTAT1 and interleukin-2 (IL-2) stimulated pSTAT5 IC_50_s. In CD4+ T cells and CD19+ B cells there were lower levels of IL-10- stimulated pSTAT3 IC50s [127].

Overall, these reports provide more supportive evidence for the use of JAK-inhibitors in JDM, but these are limited case studies with the use of several distinct JAK-inhibitors. Along with specific clinical trials of the use of JAK-inhibitors in the treatment of JDM, there is a need for standardised outcome measures for both clinical and pathological disease improvement.

## The future of JAK inhibitors

Clinical trials currently only include adult IIM patients. Successful results from these trials and validation of the case studies in JDM should be translatable to trials and treatment in juvenile disease. There are multiple JAK-inhibitors that are being trialled as potential new therapeutics for adult IIM, but these differ in their JAK targets and pharmokinetics. JAK-inhibitors provide one step further towards more targeted treatment beyond IFN blockade. It is vital to continue to investigate the exact pathogenic mechanism of the JAK/STAT pathway in IIM. If a more specific target can be found then a refined Jakinib can be developed for clinical trial in juvenile disease.

## Concluding remarks

There is a wealth of information and evidence for the potential use of JAK-inhibitors as a therapy for JDM. There is a desperate need for therapeutics that target defined pathogenic pathways in JDM. The IFN pathway is a clear point of target. JAK-inhibitors appear to be promising, but there is still the question of safety and efficacy for the use in JDM. The choice of agent will need careful consideration before choice of trials of first generation pan-JAK-inhibitors or next-generation JAK specific inhibitors. An international collaborative approach, or novel trial design for disease trials, may be required in order to achieve clear evidence of efficacy.

## Supplementary Information

**Additional file 1: Supplementary Figure 1** The role of type I IFN and the interaction with other cytokines in the immune system.

**Additional file 2: Supplementary Table 1** Metabolism, pharmacokinetics and efficacy of JAK-inhibitors.

## Data Availability

Not applicable.

## Abbreviations

JAK-STAT: Janus kinase-signal transducers and activators of transcription
JDM: Juvenile dermatomyositis
IIM: Idiopathic Inflammatory Myopathies
JDCBS: UK Juvenile Dermatomyositis cohort and biobank study
MSA: Myositis specific autoantibodies
TNFα: Tumour necrosis factor alpha
DM: Dermatomyositis
PM: Polymyositis
IFNAR: Type I IFN receptor
SLE: Systemic lupus erythematous
IFN type I: Interferon type I
pDCs: Plasmacytoid dendritic cells
IFR: IFN-regulatory factor
IRG: Interferon regulatory genes
PCR: Polymerase chain reaction
G1P2: ISG15 ubiquitin-like modifier
IRF7: Interferon regulatory factor 7
MMT8: Manual muscle testing scores
IL-6: Interleukin 6
MCP1: Monocyte chemoattractant protein 1
MCP2: Monocyte chemoattractant protein 2
IP-10: Interferon gamma-induced protein 10
TNFRII: Tumour necrosis factor receptor II
CEC: Circulating endothelial cells
SH2: Src homology domain-2
HWE: Hardy-Weinberg equilibrium
TIS: Total improvement score
MMT: Manual muscle testing-8
CMAS: Child myositis assessment scale
RA: Rheumatoid arthritis
PsA: Psoriatic arthritis
Ps: Psoriasis
AS: Ankylosing spondylitis
AA: Alopecia areata
AD: Atopic dermatitis
CD: Crohn’s disease
UC: Ulcerative colitis
Vit: Vitiligo
HPS: Hemophagocytic syndrome
GCA: Giant cell arteritis
NIU: Non-infectious uveitis
CLE: Cutaneous lupus erythematosus
LN: Lupus nephritis
DLE: Discoid lupus erythematosus
dSc: Diffuse scleroderma
