# Supplementary material for: JAK inhibitors: a potential treatment for JDM in the context of the role of interferon-driven pathology
Source: Pediatr Rheumatol Online J. 2021 Sep 25;19:146. doi: 10.1186/s12969-021-00637-8 (PMC8466894; doi:10.1186/s12969-021-00637-8)
Supplement: Supplementary file 1 — Additional file 1: Supplementary Figure 1 The role of type I IFN and the interaction with other cytokines in the immune system. [file 12969_2021_637_MOESM1_ESM.docx]

***Supplementary Figure 1* The role of type I IFN and the interaction with other cytokines in the immune system.**

Innate immune system

Adaptive immune system

This is a simplified diagram of the cells involved and cytokines produced in an autoimmune disease, highlighting the wide-reaching effects of IFN-α. Arrows indicate a major producer or target cell. B-cell activating factor (BAFF), interleukin (IL), myeloid dendritic cell (mDC), plasmacytoid dendritic cell (pDC), T-cell receptor (TCR) and tumour necrosis factor (TNF). Adapted from (128)
