# Supplementary material for: JAK inhibitors: a potential treatment for JDM in the context of the role of interferon-driven pathology
Source: Pediatr Rheumatol Online J. 2021 Sep 25;19:146. doi: 10.1186/s12969-021-00637-8 (PMC8466894; doi:10.1186/s12969-021-00637-8)
Supplement: Supplementary file 2 — Additional file 2: Supplementary Table 1 Metabolism, pharmacokinetics and efficacy of JAK-inhibitors. [file 12969_2021_637_MOESM2_ESM.docx]

***Supplementary Table 1*** Metabolism, pharmacokinetics and efficacy of JAK-inhibitors

| **Jakinib** | **Metabolism** | **Pharmacokinetics (tested in healthy adults)** | **Efficacy** |
| --- | --- | --- | --- |
| Tofacitinib | CYP 3A4 >CYCP2C19 | - Immediate release formulation HL~3h, 1h T_max,_ 93% gut availability ~30% renal excretion  - Extended release formulation HL~5.9h, 4h T_max,_ ~30% renal excretion | **RA:** phase II/III trials showed the safety and effectiveness – monotherapy and use with DMARDS(1,2)  **Ps&PsA:** effective for the use of skin and joint treatment as targets the disease associated cytokine signalling pathways(3)  **UC:** OCTAVE trials will provide data for efficacy on conclusion(4)  **CD:** unclear, possible use as maintenance therapy(5) |
| Ruxolitinib | CYP3A4 >CYP2C9 | - HL~3h, 1h T_max,_ 93% gut availability ~30% renal excretion | **RA:** Phase IIa trial showed improvement compared to placebo(6,7)  **SLE (chilblain):** Case study, patient successfully treated(8)  **DM:** Case study, patient improvement in muscle strength and skin lesions (post-polycythemia vera JAK2 V617F-positive myelofibrosis)(9)  **AA:** Improvement in patients, decreased inflammation after 12 weeks of treatment(10) |
| Baricitinib | Not CYT dependant | - HL~8h, 1.5h T_max_ post-dose_,_ ~66% renal excretion | **RA:** Phase III/IIa/b trial showed improvement(11–13)  **PP:** Phase IIb trial – improvement of PASI75 scores(14)  **Atypical neutrophilic dermatosis and AA:** improvement(15)  IIM: Phase II – pending |
| Oclacitinib | Unknown | Unknown | Treatment of canine atopic disease shows potential to translate as therapeutic strategy for allergic disease in humans(16) |

Half-life (HL); cytochrome P450 (CYP); time to maximum plasma concentration (T_max_); Extended release (XR); Plaque psoriasis (PP).

1. Conaghan PG, Østergaard M, Bowes MA, Wu C, Fuerst T, Van Der Heijde D, et al. Comparing the effects of tofacitinib, methotrexate and the combination, on bone marrow oedema, synovitis and bone erosion in methotrexate-naive, early active rheumatoid arthritis: Results of an exploratory randomised MRI study incorporating semiquantitati. Ann Rheum Dis. 2016;75(6):1024–33.

2. Genovese MC, van Vollenhoven RF, Wilkinson B, Wang L, Zwillich SH, Gruben D, et al. Switching from adalimumab to tofacitinib in the treatment of patients with rheumatoid arthritis. Arthritis Res Ther. 2016;18(1).

3. Asahina A, Etoh T, Igarashi A, Imafuku S, Saeki H, Shibasaki Y, et al. Oral tofacitinib efficacy, safety and tolerability in Japanese patients with moderate to severe plaque psoriasis and psoriatic arthritis: A randomized, double-blind, phase 3 study. J Dermatol. 2016;43(8):869–80.

4. Nielsen OH, Seidelin JB, Ainsworth M, Coskun M. Will novel oral formulations change the management of inflammatory bowel disease? Expert Opin Investig Drugs. 2016;25(6):709–18.

5. Panés J, D’Haens GR, Higgins PDR, Mele L, Moscariello M, Chan G, et al. Long-term safety and tolerability of oral tofacitinib in patients with Crohn’s disease: results from a phase 2, open-label, 48-week extension study. Aliment Pharmacol Ther. 2019;49(3):265–76.

6. Quintás-Cardama A, Kantarjian H, Cortes J, Verstovsek S. Janus kinase inhibitors for the treatment of myeloproliferative neoplasias and beyond. Nat Rev Drug Discov. 2011;10(2):127–40.

7. Shi JG, Fraczkiewicz G, Williams W V., Yeleswaram S. Predicting drug-drug interactions involving multiple mechanisms using physiologically based pharmacokinetic modeling: A case study with ruxolitinib. Clin Pharmacol Ther. 2015;97(2):177–85.

8. Wenzel J, van Holt N, Maier J, Vonnahme M, Bieber T, Wolf D. JAK1/2 Inhibitor Ruxolitinib Controls a Case of Chilblain Lupus Erythematosus. J Invest Dermatol. 2016;136(6):1281–3.

9. DeLoughery TG. The author replies. N Engl J Med. 2014;371(26):2537.

10. Xing L, Dai Z, Jabbari A, Cerise JE, Higgins CA, Gong W, et al. Alopecia areata is driven by cytotoxic T lymphocytes and is reversed by JAK inhibition. Nat Med. 2014;20(9):1043–9.

11. Keystone EC, Taylor PC, Drescher E, Schlichting DE, Beattie SD, Berclaz PY, et al. Safety and efficacy of baricitinib at 24 weeks in patients with rheumatoid arthritis who have had an inadequate response to methotrexate. Ann Rheum Dis. 2015;74(2):333–40.

12. Van Vollenhoven RF. Small molecular compounds in development for rheumatoid arthritis. Curr Opin Rheumatol. 2013;25(3):391–7.

13. Tanaka Y, Emoto K, Cai Z, Aoki T, Schlichting D, Rooney T, et al. Efficacy and safety of baricitinib in Japanese patients with active rheumatoid arthritis receiving background methotrexate therapy: A 12-week, double-blind, randomized placebo-controlled study. J Rheumatol. 2016;43(3):504–11.

14. Jabbari A, Dai Z, Xing L, Cerise JE, Ramot Y, Berkun Y, et al. Reversal of Alopecia Areata Following Treatment With the JAK1/2 Inhibitor Baricitinib. EBioMedicine. 2015;2(4):351–5.

15. Levy LL, Urban J, King BA. Treatment of recalcitrant atopic dermatitis with the oral Janus kinase inhibitor tofacitinib citrate. J Am Acad Dermatol. 2015;73(3):395–9.

16. Rönnblom L, Elkon KB. Cytokines as therapeutic targets in SLE. Nat Rev Rheumatol. 2010;6(6):339–47.
